# Testis-Specific Isoform of Na/K-ATPase (ATP1A4) Interactome in Raft and Non-Raft Membrane Fractions from Capacitated Bovine Sperm

**DOI:** 10.3390/ijms20133159

**Published:** 2019-06-28

**Authors:** Gayathri D. Rajamanickam, John P. Kastelic, Jacob C. Thundathil

**Affiliations:** 1 Department of Veterinary Clinical and Diagnostic Services, Faculty of Veterinary Medicine, University of Calgary, Calgary, AB T2N 4N1, Canada; 2 Department of Production Animal Health, Faculty of Veterinary Medicine, University of Calgary, Calgary, AB T2N 4N1, Canada

**Keywords:** bull, capacitation, proteomics, lipid rafts, Na/K-ATPase

## Abstract

The plasma membrane of sperm contains highly dynamic lipid microdomains (rafts), which house signaling proteins with a role in regulating capacitation. We reported that ATP1A4, the testis-specific isoform of Na/K-ATPase, interacted with caveolin-1, Src, epidermal growth factor receptor (EGFR) and extracellular signal-regulated kinases 1/2 (ERK1/2) in raft and non-raft domains of the plasma membrane of bovine sperm during capacitation. The objective of the present study was to use a proteomic approach to characterize the ATP1A4 interactome in rafts and non-rafts from capacitated bovine sperm. The non-raft interactome included hexokinase 1, plakophilin 1, desmoglein 1, 14-3-3 protein ζ/δ, cathepsin D and heat shock protein beta1 proteins exclusively, whereas glutathione *S*-transferase and annexin A2 were unique to raft interactome. However, a disintegrin and metalloprotease 32 (ADAM 32), histone H4, actin, acrosin, serum albumin and plakoglobin were identified in both raft and non-raft fractions of capacitated sperm. Based on gene ontology studies, these differentially interacted proteins were implicated in cell–cell adhesion, signal transduction, fertilization, metabolism, proteolysis and DNA replication, in addition to acting as transport/carrier and cytoskeletal proteins. Overall, we identified proteins not previously reported to interact with ATP1A4; furthermore, we inferred that ATP1A4 may have a role in sperm capacitation.

## 1. Introduction

Capacitation is a series of changes that sperm undergo in the female reproductive tract before they are capable of fertilization [1], including hyperactivated motility, tyrosine phosphorylation and other physiological events related to intracellular ions and initiation of signaling cascades. Although outcomes are well known, involvement of specific sperm proteins in regulating capacitation remain obscure.

As an integral membrane protein in the plasma membrane, the heterodimeric Na/K-ATPase transports 3 Na^+^ and 2 K^+^ ions between intra- and extra-cellular environments. The α subunit of Na/K-ATPase binds to ouabain (cardiotonic ligand/receptor) and is responsible for catalytic activity, whereas the β subunit is an accessory subunit and helps the α subunit to remain stable in the plasma membrane. Four distinct α subunits (α1, α2, α3 and α4) and three β subunits (β1, β2 and β3) are expressed in various cells, depending on their physiological needs [2]. ATP1A4, the testis-specific (α4) isoform of Na/K-ATPase, is responsible for most Na/K-ATPase activity in sperm, whereas some activity is also attributed to the ubiquitous (α1) isoform (ATP1A1) [3]. In addition, it is noteworthy that bovine vaginal fluid contains ouabain [4]. Based on the existence of ATP1A4 in sperm and the presence of ouabain in the female reproductive tract, we inferred that inhibition of ATP1A4 by ouabain is involved in sperm physiology.

ATP1A4 has two functions—classical and non-classical—namely, as an enzyme involved in ion transportation and a signaling molecule, respectively. Although Na/K-ATPase in raft microdomains has been regarded as being largely responsible for signaling (as it is close to other signaling molecules in the raft [5,6]), various other proteins outside raft domains (non-rafts) may have important roles in signal transduction [7]. In somatic cell studies, Na/K-ATPase signaling included both raft and non-raft domains and involved various signaling molecules, including Src, epidermal growth factor receptor (EGFR), phosphatidylinositol 3-kinase (PI3K), extracellular signal-regulated kinases 1/2 (ERK1/2) and Akt (a serine/threonine kinase) [8]. The plasma membrane of sperm has rafts and includes molecules with roles in sperm–oocyte interactions [9,10,11]. Although sperm have been reported to contain non-raft domains [12], their potential role in capacitation or other events prior to fertilization is not known.

We reported two distinct pools of ATP1A4 (raft and non-raft) which trigger specific downstream signaling molecules under ouabain-induced capacitating conditions. Specifically, using candidate proteins, we identified that ATP1A4 interacted with caveolin-1 and EGFR in the raft fraction, whereas interaction of ATP1A4 with Src, EGFR and ERK1/2 occurred in the non-raft fraction of ouabain-capacitated sperm [13]. Sperm development is inherently complex, with sperm acquiring many unique proteins, including lactate dehydrogenase (LDH-C4), sperm adhesion molecule 1 (PH-20), testis-specific isoform of angiotensin-converting enzyme (t-ACE) and testis-specific isoform of Na/K-ATPase (ATP1A4) during spermatogenesis. Accordingly, raft and non-raft membrane fractions from ouabain-capacitated sperm were subjected to immunoprecipitation–mass spectrometry (IP-MS) to characterize the interactome profile of ATP1A4. In addition, a potential role for ATP1A4 on events leading to fertilization was also proposed.

## 2. Results

### 2.1. Mass Spectrometry to Identify Partners Interacting with ATP1A4

**Figure 1:**
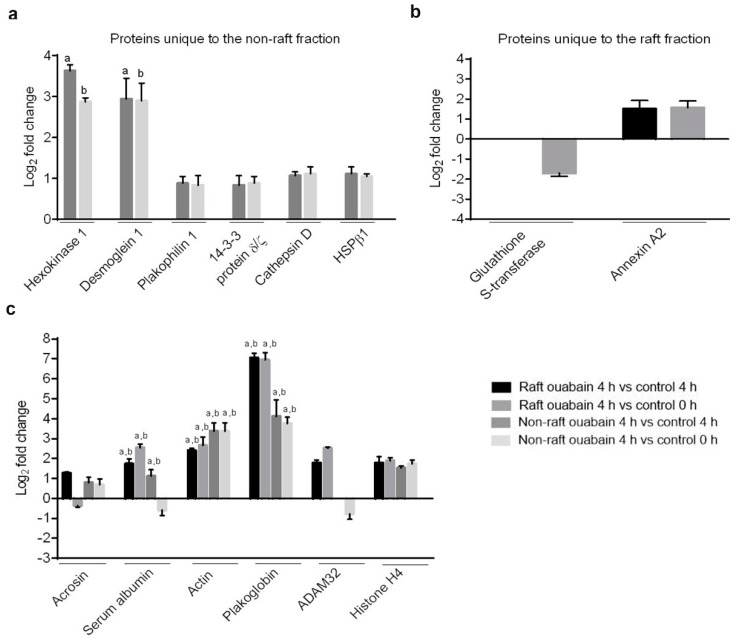
Interactome profile of ATP1A4 in raft and non-raft fractions of control (control 4 h and control 0 h) and ouabain-capacitated sperm. (**a**) Fold change of upregulated proteins that interacted with ATP1A4 in non-raft fraction. (**b**) Fold change of upregulated and downregulated proteins that interacted with ATP1A4 in raft fraction. (**c**) Fold change of upregulated and downregulated proteins identified in both fractions. ^a^ Significant (*p* < 0.05) difference compared with control 4 h group with a log_2_ FC >2; ^b^ Significant difference compared with control 0 h group with a log_2_FC >2.

Raft and non-raft fractions were isolated from control and capacitated sperm, and LC-MS/MS was used to identify ATP1A4 interactomes. Protein identities, probability scores and peptide sequences (>95% confidence level) in raft and non-raft fractions are shown in Tables S1 and S2. We identified six (non-raft) and two (raft) differentially interacted proteins (upregulated or downregulated) unique to their respective fractions (Figure 1a,b). Six proteins were common to both membrane fractions (Figure 1c). Non-raft ATP1A4 interactome comprised hexokinase 1, plakophilin 1, desmoglein 1, 14-3-3 protein ζ/δ, cathepsin D and heat shock protein β1 (HSPβ1) proteins exclusively. Glutathione S-transferase (GST) and annexin A2 were unique to the raft interactome. However, ADAM32, histone H4, actin, acrosin, serum albumin and plakoglobin were common to both raft and non-raft fractions. There were significant differences (the log_2_fold change was >2 and *p* < 0.05) for hexokinase 1, actin, desmoglein 1, serum albumin and plakoglobin, which were significantly upregulated in their interactions with ATP1A4 in ouabain-capacitated sperm compared to their respective controls (Figure 1a,c).

### 2.2. Ontology and Pathway Analysis of ATP1A4 Interactome in Raft and Non-Raft Fractions

**Figure 2:**
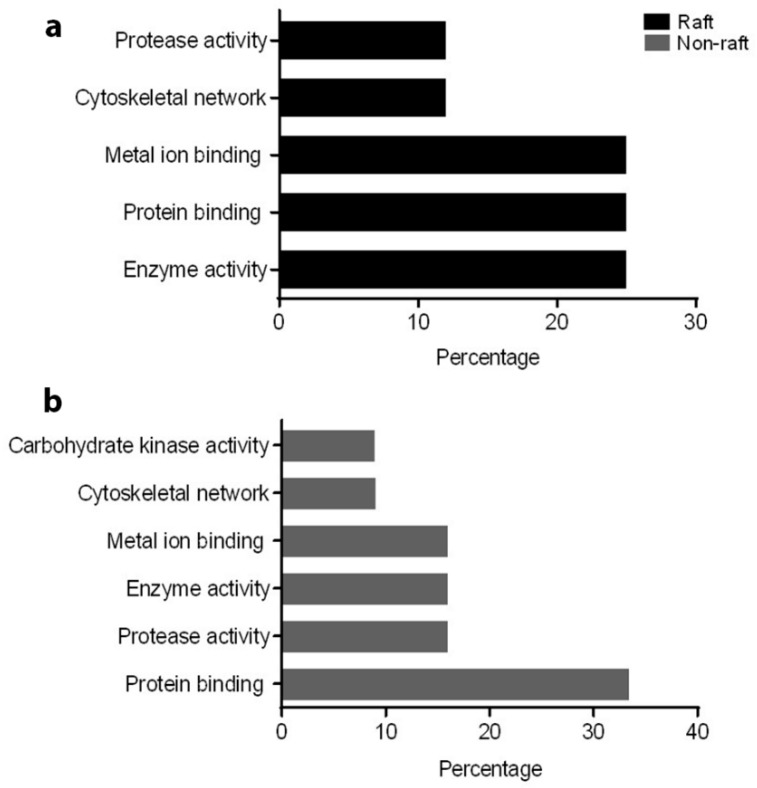
Percentage contribution of molecular functions of differentially interacted proteins in (**a**) raft and (**b**) non-raft membrane fractions.

**Figure 3:**
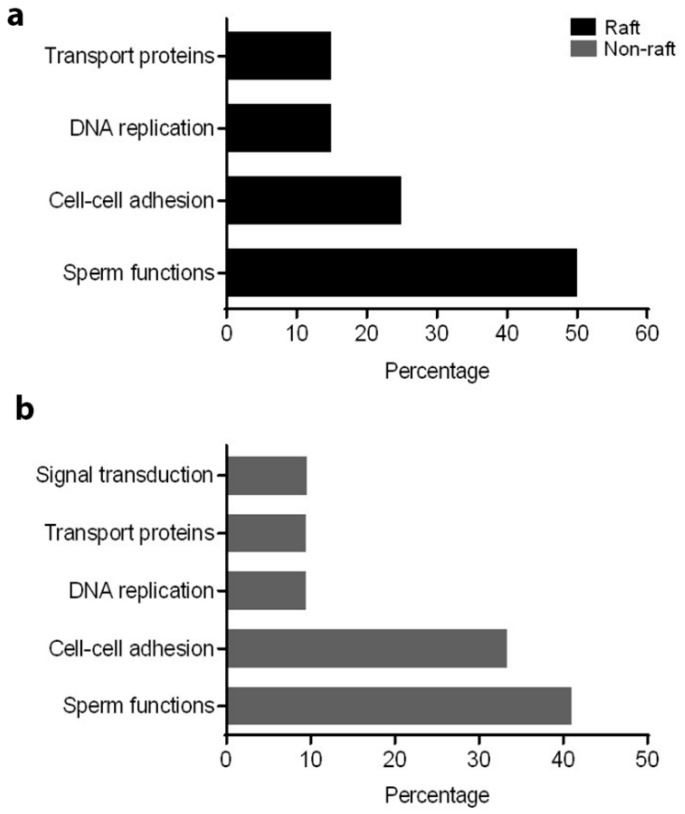
Percentage contribution of biological processes of differentially interacted proteins in (**a**) raft and (**b**) non-raft membrane fractions.

**Figure 4:**
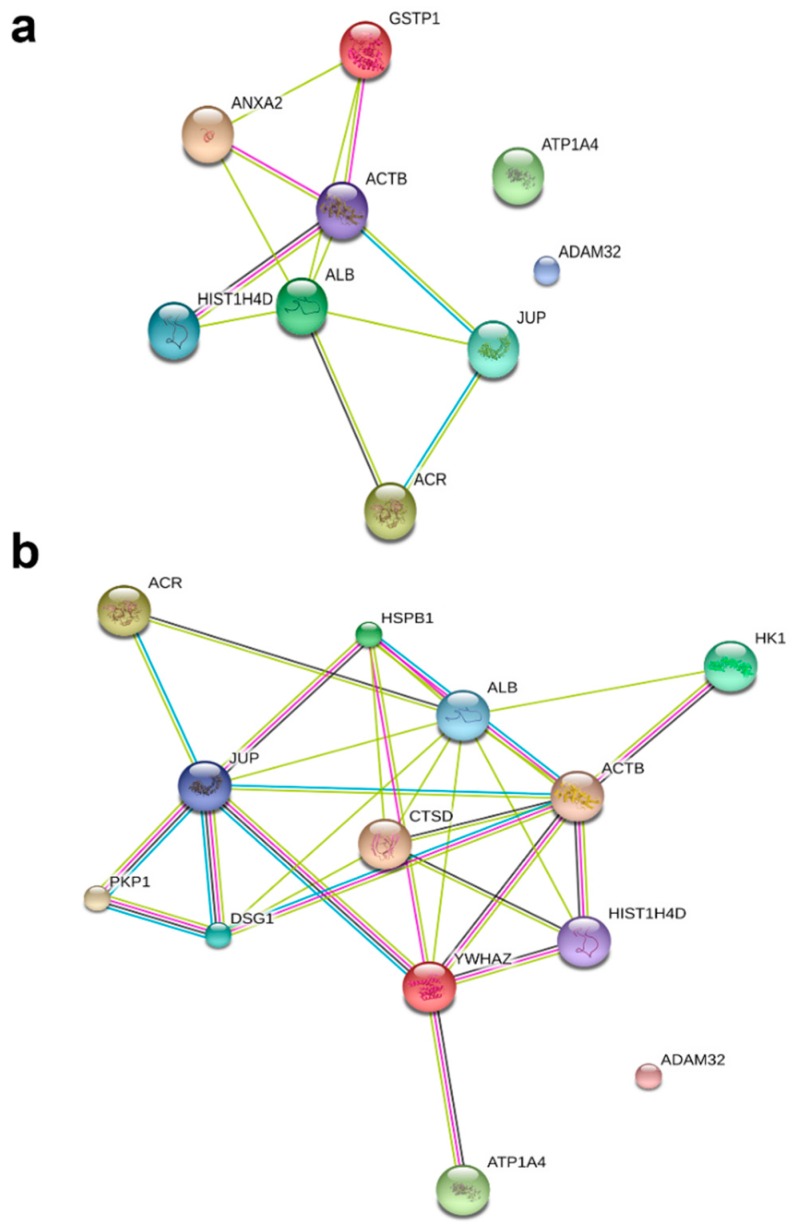
Protein–protein interaction (PPI) analysis using STRING. (**a**) and (**b**) represent PPI network analyses from raft and non-raft fractions, respectively, based on evidence and confidence level. More interconnecting lines indicate a strong evidence for PPI and different colours indicate the source of evidence between interacting proteins. For example, black line represents co-expression studies; pink line denotes experimentally determined interactions; green line suggests curated databases; and blue line suggests literature mining. The proteins identified in raft and non-raft fraction clusters are shown in Table 1.

Based on PANTHER analysis, protein binding was a major classification in molecular function for both raft (25%) and non-raft (33.33%) fractions. However, among rafts, enzyme activity and metal ion binding had equivalent contributions (25% each) to that of protein binding (Figure 2a,b). Within non-rafts, enzyme activity, protease function and metal ion binding were the second major contributors (16% for each category) to molecular function. Regarding biological process, sperm functions (motility, fertilization) were a major category in raft (50%) and non-raft (41%) fractions. Proteins involved in cell–cell adhesion contributed 25% and 33.33% to biological processes in the raft and non-raft fractions, respectively (Figure 3a,b). Using STRING network analysis, the interface displayed colour-coded edges between proteins, based on evidence from literature mining, curated databases, experimental/biochemical data, co-expression and co-occurrence across genomes. In both fractions, most proteins were connected by at least two interconnecting lines, indicating the strength of evidence for protein–protein interaction (PPI). Although most proteins were connected, ADAM32 could not be linked to any identified protein in the network, in either raft or non-raft fractions. Furthermore, ATP1A4 was not connected to the network in the raft fraction (Figure 4a,b).

### 2.3. Validation of Mass Spectrometry Data for Selected Candidate Proteins

**Figure 5:**
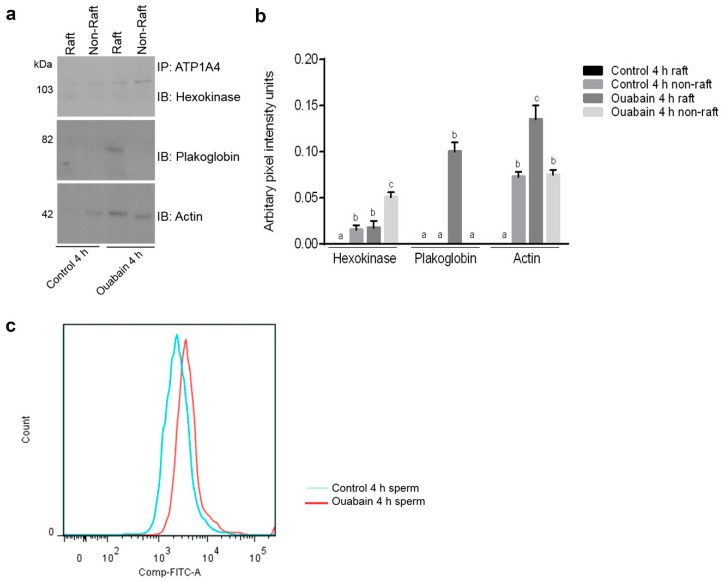
Validation of selected proteins that interacted with ATP1A4 during ouabain-induced capacitation. (**a**) Solubilized preparations of raft and non-raft membrane fractions were immunoprecipitated for 12 h with a cross-linked ATP1A4 antibody–protein A bead slurry and subsequently immunoblotted with hexokinase, plakoglobin and actin antibodies. (**b**) Protein pixel intensities (arbitrary units) were determined using Adobe Photoshop CS6. (**c**) FITC-phalloidin was used to label F-actin formation in capacitated sperm and flow cytometry was used to determine fluorescence; a histogram of fluorescence intensity is shown. Data were expressed as mean ± SEM (*n* = 3). ^a–c^ Values without a common letter differed (*p* < 0.05).

**Figure 6:**
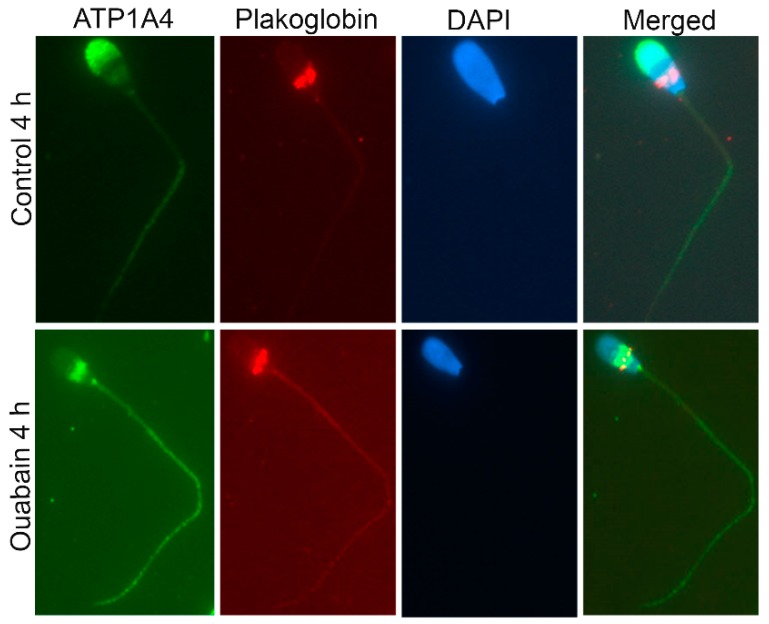
Co-localisation of ATP1A4–plakoglobin during sperm capacitation. Representative images (60×) of (green) ATP1A4, (red) plakoglobin, (blue) nuclei and merged plakoglobin, ATP1A4 and DAPI staining in control 4 h and ouabain 4 h capacitated sperm.

Since ouabain-capacitated sperm had upregulated and significant interactions compared to both control groups (control 0 h and 4 h), only one control (control 4 h) group was used for subsequent validation. Hexokinase 1, plakoglobin and actin, with significant differences in interaction between control and ouabain-capacitated sperm, were designated for validation. Immunoprecipitation experiments indicated that hexokinase was more prominent in the non-raft fraction of ouabain-capacitated sperm compared to its interaction in control sperm (Figure 5a,b). Plakoglobin was present only in the raft fraction of ouabain-capacitated sperm (Figure 5a,b), with no indications of interaction in control sperm. Microscopically, plakoglobin was restricted to the equatorial segment, whereas ATP1A4 was restricted to the anterior acrosome in control sperm (Figure 6). However, in ouabain-capacitated sperm, ATP1A4 signal translocated to the equatorial segment and post-acrosome regions and merged with plakoglobin signal in the equatorial segment. (Figure 6). There was more actin in raft versus non-raft fractions of ouabain-capacitated sperm (Figure 5a,b). Based on FITC-phalloidin fluorescence to detect F-actin formation (flow cytometry), the histogram corresponding to ouabain-capacitated sperm (red) was pushed more towards the right on the FITC log scale in the *x*-axis compared to the histogram from control sperm (blue; Figure 5c).

## 3. Discussion

A proteomic approach was used to investigate profiles of ATP1A4-interacting partners in raft and non-raft membrane fractions during capacitation. Hexokinase 1, plakophilin-1, 14-3-3 protein ζ/δ, cathepsin D, desmoglein 1 and HSPβ1 comprised the non-raft interactome, whereas GST and annexin A2 were present only in the raft fraction. Both raft and non-raft fractions contained ADAM 32, histone H4, actin, acrosin, serum albumin and plakoglobin. We speculate that these differentially interacted proteins may be involved in cell–cell adhesion, fertilization, signal transduction, metabolism, DNA replication, proteolysis, and furthermore, they may have roles as transport/carrier and cytoskeletal proteins. Validation confirmed that plakoglobin interacted with ATP1A4 in rafts of ouabain-capacitated sperm, and potentially co-localised with ATP1A4 in the equatorial segment. There was interaction between ATP1A4 and hexokinase in the non-raft fraction, whereas interaction with actin was greater in the raft fraction and the most F-actin fluorescence was present in sperm capacitated with ouabain.

Label-free proteomic investigations (e.g., spectral count) are becoming more accepted as semi-quantitative approaches to assess protein abundance; they have been highly correlated with relative protein abundance and have a dynamic range greater than two orders of magnitude [14,15]. In this study, spectral count was noticed for certain signaling molecules from sperm incubated under control (4 h) conditions. Control (4 h) media contains BSA, calcium and bicarbonate which promote capacitation and therefore, sperm incubated in this media always display a certain degree of capacitation-associated physiological changes [16] which is manifested by its interaction with signaling molecules. Despite the presence of the above-mentioned ions/molecules, sperm requires a capacitating agent (such as ouabain) in the media to become fully capacitated [16,17]. We believe that the presence of ouabain enhances interactions between ATP1A4 and signaling molecules (compared to control 4 h media), which is manifested by the differences in the log fold change (Table 1) of their respective spectral count.

To understand the role of these identified proteins in their interaction with ATP1A4, it is imperative to deduce the function of these individual proteins during capacitation. In that regard, PANTHER ontology tool provided biologically relevant information about different proteins that were involved in ATP1A4 interaction. Proteins involved in various categories of molecular function such as enzyme activity (ADAM32), signal transduction (14-3-3 ζ/δ), protein binding (plakoglobin), carbohydrate kinase activity (hexokinase 1) and cytoskeleton (actin), which are involved in biological processes like fertilization, signal transduction, cell–cell adhesion, metabolism and motility, respectively, were identified. It was noteworthy that ATP1A4 and ADAM32 could not be linked to the remainder of the identified proteins using STRING pathway. Perhaps these proteins have not been studied very much together and therefore their interactions are not recognized by STRING, suggesting that the identified network was highly novel. 

**Figure 7:**
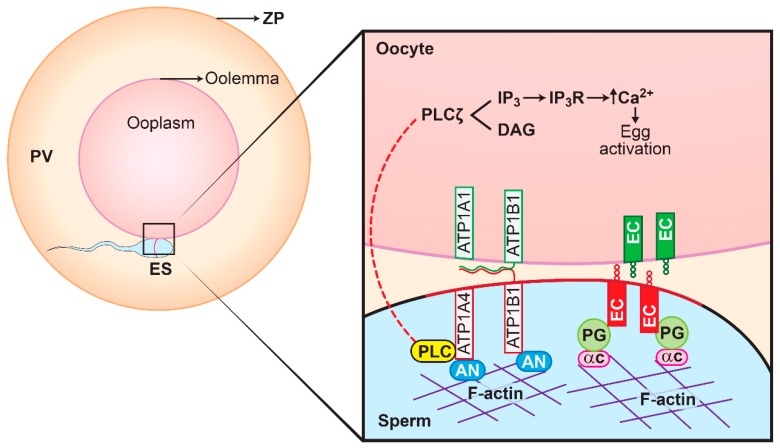
Proposed model of plakoglobin, α and β subunits of ATP1A4, E-cadherin during sperm–oocyte adhesion and fusion. Complementary E-cadherin molecules on sperm and oocyte may bind, augmented by E-cadherin (EC) binding to the plakoglobin (PG)–α-catenin (αC)–F-actin network. ATP1A4 possibly binds to ankyrin (AN), an anchor protein, causing it to interact with the actin cytoskeleton and thereby linking ATP1A4 to the F-actin–plakoglobin–E-cadherin complex. Concurrently, ATP1A4 interaction with PLCζ (sperm oocyte activating factor) [22] would promote entry of PLCζ from sperm (broken arrows), thereby increasing release of intracellular calcium and re-starting meiosis in the oocyte. Furthermore, ATP1A4 may promote sperm–oocyte interactions by complementary binding of the *N*-glycan motifs of the respective β subunits of Na/K-ATPase (ATP1B1/ATP1B3) that are in present in the gametes. This figure is reproduced with permission from Jacob C. Thundathil, Gayathri D. Rajamanickam and John P. Kastelic’s review article on “Na/K-ATPase and regulation of sperm function” published in the Proceedings of the 10th International Ruminant Reproduction Symposium hosted by the Brazilian College of Animal Reproduction, Brazil 2018.

A few proteins (hexokinase, actin and plakoglobin) that are relevant in the broader context of ATP1A4 role and its physiology during capacitation/fertilization were subsequently selected for functional validation. Extracellularly, E-cadherins on adjacent epithelial cells promote cell-to-cell adhesion, whereas intracellularly they are linked to actin via the catenins (plakoglobin) [18]. Catenin proteins and E-cadherin are expressed in oocytes and sperm, where they are specifically localised to the microvillar region on the oolemma and the equatorial segment (ES) of the sperm head, respectively [19,20]. If gamete interactions (fusion between ES on sperm head and microvilli of the oolemma) involve mechanisms that are identical to epithelial cell–cell adhesion, it is likely that E-cadherins and plakoglobin have roles in sperm–oocyte adhesion and fusion, based on their locations in gametes. Furthermore, it is possible that the N-glycan motif of the β1 and β3 subunits of Na/K-ATPase in sperm would bind to its corresponding β subunit in the oocyte, thereby promoting adhesion without involvement of the E-cadherin pathway [21]. A proposed model involving plakoglobin, ATP1A4 (both α and β subunits) and E-cadherin is presented in Figure 7.

In sperm, compartmentalised glycolysis occurs instead of ATP diffusing along the flagellum [23]. For example, hexokinase which catalyses the initial step in glycolysis is present in the acrosome and could provide a local source of ATP required by ATP1A4 to function as an enzyme [24]. In HeLa cells, triose-phosphate isomerase, a member of the glycolytic pathway interacts with phosphorylated cofilin (an actin binding protein), which in turn binds with the cytoplasmic domain of Na/KATPase [25]. It is likely that ATP1A4, present in the sperm head, interacts with hexokinase via cofilin or other actin-binding proteins. 

The 14-3-3 (YWHA) family of proteins function as adaptor proteins and have a role in various physiological events, including cytoskeletal rearrangements, metabolism, cell cycle control, apoptosis, stress response and gene expression [26]. YWHA (14-3-3) protein interacts with phosphorylated Na/K-ATPase α subunit in opossum kidney (OK) cells, creating a site for binding of PI3K to the N-terminus of the phosphorylated α subunit [27]. In the present study, PI3K was a significant hit, but was not implicated as a differentially interacting protein, as it failed to meet cut-offs for identification of peptides and proteins. In sperm, PI3K promotes formation of PIP_3_ (phosphatidylinositol 3,4,5-triphosphate) from PIP_2_ (phosphatidylinositol 4,5-bisphosphate); the latter is a cofactor for PLD (phospholipase D) activation and also promotes formation of F-actin (filamentous actin) [28]. F-actin acts as a scaffold between the plasma membrane and outer acrosomal membrane in sperm, thereby immobilizing molecules that are involved in oocyte activation [29]. Similar to the YWHA family, annexins (calcium-dependent phospholipid-binding proteins) have high PIP_2_ affinity and link Na/K-ATPase and actin [30,31]. 

It was noteworthy that we detected an interaction between ATP1A4 and proteases (cathepsin D). There was considerable ATP1A4 fluorescence in the ES and postacrosomal regions versus the anterior acrosomal region following capacitation. The underlying mechanism is not understood but may be due to ATP1A4 translocation from the anterior acrosomal region to the ES and postacrosomal areas. Presumably this would involve ATP1A4 being freed from its cytoskeletal anchorage. Calpain can cause hydrolysis of several proteins, including spectrin and ankyrin [32]; those two are known to bind ATP1A4 and attach it to the actin cytoskeleton. Perhaps similar proteolytic mechanisms could function during capacitation that is responsible for ATP1A4 redistribution via cathepsins. 

Heat shock proteins (HSPs; chaperones) are known to be involved in de novo folding of proteins, assembling multiprotein complexes, preventing aggregation of protein [33,34] and delivering proteins (protein trafficking) to intracellular domains. For example, HSP 70 attaches to Na/K-ATPase and promotes detachment from the cytoskeleton after renal injury [35]. Perhaps HSPs mediate relocation of ATP1A4 from the anterior acrosome in uncapacitated sperm to the ES and postacrosomal region during capacitation. 

## 4. Materials and Methods

### 4.1. Semen Collection and In Vitro Capacitation of Bovine Sperm 

This study was approved by the University of Calgary Institutional Animal Care and Use Committee (protocol number AC13-0147). An artificial vagina was used to collect semen from mature Holstein bulls. The semen was diluted 1:1 with TALPH ([16] and then placed in a thermos to maintain it at approximately 35 °C prior to arrival at the laboratory. Diluted semen was placed on top of a two-layer percoll gradient (45–90%) and centrifuged at 700 *g* for 30 min (25 °C). The pellet was resuspended in TALP (Tyrode’s-bicarbonate buffered medium), with percoll removed by centrifugation at 380 *g* for 10 min at 25 °C. Sperm concentration was determined with a hemocytometer, with TALP added to reach a concentration of 200 × 10^6^ sperm/mL. Ouabain was dissolved in Sp-TALP to form a stock solution (100 µM) that was kept at 4 °C. As needed, this was diluted in complete Sp-TALP medium (TALP with 1 mM pyruvate, 25 mM NaHCO_3_, 2 mM Ca^2+^) to create an ouabain working solution (50 nM). Two control groups were used: fresh uncapacitated sperm (control 0 h) and sperm incubated in Sp-TALP for 4 h (control 4 h). Both control 4 h group and ouabain 4 h group were incubated for 4 h at 39 °C under high humidity. Treatment with 50 nM ouabain consistently induced capacitation, manifested by hyperactivation and tyrosine phosphorylation of several sperm proteins [36,37].

### 4.2. Isolation of Raft and Non-Raft Fractions from Uncapacitated and Capacitated Sperm

Raft and non-raft membrane fractions were isolated from fresh uncapacitated and capacitated sperm, using sodium carbonate as described [13]. The non-detergent lysis buffer contained 0.5 M Na_2_CO_3_ (pH 11), 1 mM EDTA, 1 mM Na_3_VO_4_, 1 mM NaF and various protease inhibitors (Roche, Laval, QC, Canada), One billion (1 × 10^9^) sperm was added to the pre-chilled lysis buffer, homogenized and maintained on ice for 30 min. Centrifugation (1000× *g*, 5 min) was done to pellet sperm and the supernatant was mixed 1:1 with 80% sucrose (*w*/*v*) in MBS (MES buffered saline) buffer (25 mM MES (2-*N*-morpholino ethanesulfonic acid), 150 mM NaCl, pH 6.5). The resulting mixture was placed at the bottom, followed by 35% sucrose in the middle and 5% sucrose at the top of the centrifugation tube. Tubes were centrifuged at 110,000 *g* for 12 h at 4 °C (SW 41 Ti Rotor, Beckman Coulter Inc., Brea, CA, USA), resulting in rafts forming an opalescent band at the interface of 5–30% sucrose. In contrast, non-rafts accumulated at the lowermost part of the gradient. Successful separation of raft and non-raft fractions is demonstrated in Figure S1. To remove sucrose, both fractions were subjected to ultra-centrifugation (189,000 *g* for 1 h) in a TLA 100.3 rotor (Beckman Coulter Inc.), forming pellets that were used in our studies. 

### 4.3. Immunoprecipitation and SDS-PAGE

Pelleted raft and non-raft fractions from uncapacitated and capacitated sperm were lysed in ice-cold buffer containing 150 mM NaCl, 10 mM Tris·HCl (pH 8.0), 1% Triton X-100, 60 mM octylglucoside, 1× protease inhibitor cocktail, 1 mM NaF and 1 mM Na_3_Vo_4_ for 30 mins. Preclearing was done with protein A beads for 30 min at 4 °C on a rocker. Cross-linking was done using ATP1A4 antibody (3 µg/mL) incubated with the protein-A bead slurry for 1 h on a rocker at 4 °C, in accordance with manufacturer’s instructions (Thermo Fisher Scientific, Missisauga, ON, Canada. Beads were washed to remove unbound antibody, followed by incubation in conjugation buffer (20 mM Na_3_PO_4_, pH 7.4) containing BS3 (cross-linker) for 30 min at RT. Termination of cross-linking was done by quenching with 1 M Tris, pH 7.5 for 15 min at RT. Precleared supernatant from raft and non-raft fractions of control and capacitated groups were incubated with cross-linked antibody–protein A bead slurry for 12 h at 4 °C on a rocker. Thereafter, beads were centrifuged (500 *g* for 1 min at 4 °C) and washed (3×) in PBS (phosphate buffered saline) with 0.1% Tween-20. Glycine (100 mM, pH 2.5) was added, with gentle rocking at RT for 10 min, to elute proteins from beads. Elution was repeated and proteins were separated on SDS-PAGE and stained with 0.1% Coomassie Brilliant Blue R-250 (50% methanol/10% acetic acid/40% water) for 2 h at RT. Destaining of the gel was done with fresh 50% methanol/10% acetic acid/40% water. At 20 min intervals, the gel was examined. Once faint bands were apparent, the gel was cleared by washing with Milli-Q water.

### 4.4. Protein Digestion

All steps involved in protein isolation and identification were done at the mass spectrometric facility, SAMS Centre, University of Calgary. Coomassie-stained protein gel bands were cut into approximately 1 mm^3^ pieces, resuspended in 50 mM ammonium bicarbonate/acetonitrile (50:50, *v*/*v*), reduced with 10 mM dithiothreitol for 30 min at 56 °C and alkylated with iodoacetamide for 30 min in the dark at RT. Following trypsin digestion at 16 h at 37 °C, the supernatant was placed into a tube with 5 µL of acidifying solution (60% acetonitrile, 30% water and 10% trifluoroacetic acid) and samples lyophilized and resuspended in 10 µL of 1% formic acid in water.

### 4.5. LC-MS/MS Analysis

Liquid chromatography (LC; Agilent 1260 Infinity chip cube interface) tandem mass spectrometry (MS/MS) was done on an Agilent 6550 iFunnel quadrupole–time-of-flight (Q-TOF) mass spectrometer (Agilent Technologies, Mississauga, ON, Canada) to analyze tryptic peptides. MassHunter (version B.05.00) was used to control the LC and Q-TOF. Samples were loaded with a capillary pump using A1 (97% water, 2.9% acetonitrile, 0.1% formic acid) and B1 (90% acetonitrile, 9.9% water; 0.1% formic acid) solutions. The gradient for peptide elution was generated with a nanopump that used A1 (97% water, 2.9% acetonitrile, 0.1% formic acid) and B1 (97% acetonitrile, 2.9% water; 0.1% formic acid) solutions. Aliquots (1 µL) of tryptic peptides were put into a C18 trap column of an Agilent chip, in enrichment mode (2.5 µL/min flow rate and 3% B1). After loading the sample into the enrichment column, the chip valve was switched from enrichment to analysis mode. Peptides were eluted with a 25 min linear gradient from 3% to 50% B1 generated by the nanopump (0.3 µL/min). Peptides were electrosprayed into the Q-TOF which was operated in positive auto MS/MS mode. Precursor ions with an m/z comprised between 275 and 1700 were acquired at a scan rate of 250 ms/spectrum, and the 10 most abundant precursors for each cycle having a charge higher than 1, an intensity of at least 1000 counts and a peptidic isotopic model fragmented by collision-induced dissociation (CID) were identified. Fragment ions with an *m*/*z* from 50 to 1700 were acquired at a scan rate of 333.3 ms/spectrum.

### 4.6. Database Search and Criteria for Protein Identification

Data files from LC/MS-MS data files (*.d file) were imported into Agilent MassHunter qualitative analysis software (version B.05.00), converted into a Mascot Generic Format (MGF) file using default parameters, and this file was used to search the UniProt database using the Mascot algorithm (Matrix Sciences, version 2.4). Search parameters for the MS data included *Bos taurus* taxonomy, trypsin as enzyme, a maximum number of missed cleavage of 1, cysteine carbamidomethylation as fixed modification, methionine oxidation as variable modification and a mass error tolerance of 0.2 Da for both the MS and MS/MS data. Only peptides with a Mascot expectation value <0.05 and Mascot ion score of 23 were retained and subjected to further analysis. Identified peptides and proteins were validated with Scaffold 4 (Proteome Software Inc., Portland, OR, USA). Protein identifications were accepted provided they demonstrated >95% probability and they had to contain a minimum of two identified peptides. Common contaminants (e.g., keratins and trypsin) were manually excluded. Normalized spectral count (NSC) was obtained for each identified protein; it was calculated based on the number of spectra assigned to a protein and subsequently multiplied by the average spectral counts of all proteins in three bulls, divided by the spectral counts of total proteins in a particular bull sample (Table 1).

### 4.7. Gene Ontology and Pathway Analysis

Gene ontology was obtained using PANTHER (http://www.pantherdb.org/) to classify differentially interacted proteins based on molecular function (MF) and biological processes (BP). The protein–protein interaction network was determined with STRING version 10.5 (https://string-db.org/). UniProt accession numbers were submitted for all identified proteins and mapped against the *Bos taurus* database. Interaction network was obtained based on confidence level and evidence.

### 4.8. Western Blotting

Immunoprecipitates eluted from raft and non-raft fractions of control 4 h and ouabain-capacitated sperm were separated on 10% SDS-PAGE gels and electrotransferred to nitrocellulose membranes. Gels were blocked with 3% (*w*/*v*) skim milk in TTBS (Tween 20-Tris-based saline) for 1 h, then incubated overnight at 4 °C with the following antibodies: hexokinase 1 (1:2000; Novus Biologicals, Oakville, ON, Canada), plakoglobin (1:100; Sigma-Aldrich, Oakville, ON, Canada), actin (1:500; Santa Cruz Biotechnology, Dallas, TX, USA). After washing membranes three times in TTBS for 10 min, they were incubated with respective HRP conjugated goat anti-rabbit and anti-mouse IgG (1:5000) for 1 h at RT. Following washing, chemiluminescence was used to detect immunoreactive bands.

### 4.9. Co-Localisation of Plakoglobin with ATP1A4 in Capacitated Sperm

Control 4 h and ouabain-capacitated sperm were adhered onto poly-l-lysine coated slides, fixed with 2.5% paraformaldehyde (PFA) for 15 min, permeabilized with 0.2% Tween-20 for 10 min at RT, washed in PBS and incubated with 10% serum for blocking (species of origin used was dictated by source of secondary antibodies) for 1 h at RT. Co-incubation of ATP1A4 (1:100; custom-made at the University of Calgary, Calgary, AB, Canada) with plakoglobin (1:20) was performed in 1% serum overnight at 4 °C. Following washing in PBS (5×), chicken anti-rabbit alexa 488 (ATP1A4) and goat anti-mouse Cy3 (plakoglobin) secondary antibodies were added at 1:200 for 1 h at RT. Cells were washed in PBS and mounted with Vectashield (Vector Laboratories, Burlingame, CA, USA) containing DAPI and stored at –20 °C. Images were captured using a Zeiss AxioVert.A1 inverted fluorescence microscope and Axiocam ICc 5 camera (Carl Zeiss Microscopy GmBH, Munich, Germany).

### 4.10. Flow Cytometric Analysis of F-actin in Sperm

F-actin filaments were stained in accordance with manufacturer’s instructions (Thermo Fisher Scientific, Mississauga, ON, Canada). Following capacitation, control 4 h and ouabain-capacitated sperm were washed with PBS, to which 1 µL of Fixable Live and Dead Cell Stain (Thermo Fisher Scientific) was added, and subsequently incubated for 30 min at RT. Sperm were fixed by addition of 4% PFA for 10 min, permeabilized with 0.1% Triton X-100 for 5 min and incubated in PBS containing 1% BSA and FITC-phalloidin (0.33 µM; Thermo Fisher Scientific) for 1 h at 37 °C. After washing, data acquisition was done with an Attune Acoustic Focusing Cytometer (BD Biosciences, Mississauga, ON, Canada). A 488 nm laser for FITC and a 405 nm laser for the fixable live and dead (violet) dye were used as the excitation source. Voltage settings used were as follows: FSC—1250, SSC—1650, FITC—1500, violet—1650. Negative and autofluorescent controls (cells only) were done to establish voltages and gates, whereas single colour controls (violet alone and FITC per se) were used for compensation to minimise violet fluorescence being detected in the FITC channel. Subsequently, detector 1 (emission range, 450 ± 20 nm) was used for detecting violet fluorescence (viability) and detector 2 (emission range, 530 ± 15 nm) was used to detect green (FITC-phalloidin) fluorescence. Overall, 20 × 10^3^ events were captured in a scatter plot and histogram.

### 4.11. Statistical Analysis

Three bulls provided ejaculates and analysis was done with log_2_ values of mean normalized spectral counts of identified proteins from raft and non-raft fractions of control (control 0 h and 4 h) and ouabain-capacitated sperm. Expression levels of some proteins were zero; therefore, fold change was calculated as the difference between the log_2_ values of spectral counts between control and ouabain-capacitated sperm. Fold change and pixel intensity values were analysed by ANOVA, followed by multiple comparison post-hoc tests. All statistical analyses were done with GraphPad Prism version 7.00 (GraphPad Software, La Jolla, CA, USA) and *p* < 0.05 was considered significant.

## 5. Conclusions

To our knowledge, this was the first comprehensive report on ATP1A4 interactome from capacitated bovine sperm. Although physiological relevance of these interactions requires future studies, these data demonstrate the dynamic organisation of ATP1A4 interactome being modulated by ouabain and opens new areas of investigation by which ATP1A4 influences sperm biology. 

## Figures and Tables

**Table 1:** Average normalised spectral counts of proteins identified in control and ouabain-capacitated sperm.

	**Raft Interactome**							
**Accession #**	**Protein**	**Gene name**	**Control 0 h**		**Control 4 h**		**Ouabain 4 h**	
			**Mean**	**SD**	**Mean**	**SD**	**Mean**	**SD**
P79343	Acrosin	*ACR*	2.5	1.4	0	0	2	0.7
P02769	Serum albumin	*ALB*	0	0	0	0	1.5	0.8
P60712	Actin, cytoplasmic	*ACTB*	2.5	0.9	0	0	4.5	1.5
Q8SPJ1	Junction plakoglobin	*JUP*	0	0	0	0	11	1.4
Q2NKZ3	ADAM32	*ADAM32*	0	0	1.5	1.2	5.5	1.5
E1BBP7	Histone H4	*HIST1H4D*	0	0	0	0	4	1.8
Q3SZU6	Glutathione S-transferase	*GSTP1*	3.5	1.2	0	0	0	0
P04272	Annexin A2	*ANXA2*	0	0	0	0	3.5	1.7
	**Non-Raft Interactome**							
**Accession #**	**Protein**	**Gene name**	**Control 0 h**		**Control 4 h**		**Ouabain 4 h**	
			**Mean**	**SD**	**Mean**	**SD**	**Mean**	**SD**
P79343	Acrosin	*ACR*	6.5	2.5	6	3.4	9	3.4
Q5W5U3	Hexokinase	*HK1*	1.5	1.2	0	0	11.5	2.1
P60712	Actin, cytoplasmic 1	*ACTB*	0	0	0	0	8.5	2.5
Q8SPJ1	Junction plakoglobin	*JUP*	0	0	0	0	12	2.4
E1BBP7	Histone H4	*HIST1H4D*	0	0	0	0	3	1.4
Q28161	Plakophilin-1	*PKP1*	0	0	0	0	2	0.4
P63103	14-3-3 protein zeta/delta	*YWHAZ*	0	0	0	0	2	0.4
P80209	Cathepsin D	*CTSD*	0	0	0	0	2	0.4
E1BEL7	Heat shock protein beta-1	*HSPB1*	0	0	0	0	2	0.4
Q2NKZ3	ADAM32	*ADAM32*	1.5	0.9	0	0	0	0
Q03763	Desmoglein 1	*DSG1*	0	0	0	0	2	0.7
P02769	Serum albumin	*ALB*	8.5	2.1	3.5	1.8	6.5	1.7

## Supplementary Materials

Supplementary materials can be found at https://www.mdpi.com/1422-0067/20/13/3159/s1.
