# Supplementary material for: Testis-Specific Isoform of Na/K-ATPase (ATP1A4) Interactome in Raft and Non-Raft Membrane Fractions from Capacitated Bovine Sperm
Source: Int J Mol Sci. 2019 Jun 28;20(13):3159. doi: 10.3390/ijms20133159 (PMC6651793; doi:10.3390/ijms20133159)
Supplement: Supplementary file 1 [file ijms-20-03159-s001.pdf]

Supplementary Table 1: Protein identities, their probability scores (protein score and expect score) and peptide sequences (>95 % confidence) in the non-raft fraction.

Protein name	Protein score	Expect score	Peptide sequences
Glial fibrillary acidic protein	97	0.000058	LALDIEIATYR
		0.0000026	LALDIEIATYR
Phosphatidylinositol 3-kinase	25	0.038	HGDDLR
Uveal autoantigen with coiled-coil domains and ankyrin repeats protein	30	0.03	TEELNR
ADAM metallopeptidase domain 32	347	0.0072	SGSICDK
		0.0059	YTFCPWR
		0.00028	CSEVGPYINR
		0.0073	DSASVIYAFVR
		0.00044	DSASVIYAFVR
		0.00047	LICTYPLQTPFLR
		0.0039	LICTYPLQTPFLR
		0.001	LICTYPLQTPFLR
		0.0000038	AYCFDGGCQDIDAR
		0.00000043	AYCFDGGCQDIDAR
		0.0000042	VNQCSAQCGGNGVCTSR
		0.0000048	NAPFACYEEIQSQTDR
		0.000019	NAPFACYEEIQSQTDR
Alpha-fetoprotein	24	0.0093	YIYEIAR
Junction plakoglobin	214	0.011	ATIGLIR
		0.0038	LVQLLVK
		0.000043	EGLLAIFK
		0.00027	QEGLESVLK
		0.000085	TMQNTSDLDTAR
		0.000046	ALMGSPQLVAAVVR
		0.01	LLNQPNQWPLVK
		0.01	NEGTATYAAAVLFR
		0.004	NLALCPANHAPLQEAAVIPR
		0.0007	VLSVCPSNKPAIVEAGGMQALGK
		0.00086	ILVNQLSVDDVNVLTCATGTLSNLTCNNSK
Catenin beta-1	54	0.000043	EGLLAIFK
Lysozyme	89	0.0026	STDYGIFQINSR
		0.00051	STDYGIFQINSR

		0.0000052	STDYGIFQINSR
Annexin A2	72	0.0036	QDIAFAYQR
	72	0.0000043	TNQELQEINR
Actin, cytoplasmic	61	0.023	IIAPPER
		0.021	IIAPPER
		0.0044	AGFAGDDAPR
		0.013	EITALAPSTMK
		0.0013	LCYVALDFEQEMATAASSSSLEK
		0.023	IIAPPER
		0.021	IIAPPER
		0.0044	AGFAGDDAPR
		0.0013	LCYVALDFEQEMATAASSSSLEK
		0.023	IIAPPER
		0.021	IIAPPER
		0.0044	AGFAGDDAPR
		0.013	EITALAPSTMK
ACTA2 protein	35	0.0044	AGFAGDDAPR
		0.013	EITALAPSTMK
Similar to beta actin (Fragment)	31	0.0013	LCYVALDFEQEMATAASSSSLEK
Actin (Fragment)	30	0.023	IIAPPER
		0.021	IIAPPER
Heat shock protein beta-1	59	0.000009	LFDQAFGLPR
Hexokinase	58	0.00011	SANLVAATLGAILSR
		0.0014	SANLVAATLGAILSR
Desmoglein-1	53	0.000012	IHSDCAANQQVTYR
Glutathione peroxidase 7	26	0.042	AVNIR
14-3-3 protein sigma	38	0.0045	NLLSVAYK
		0.0047	DSTLIMQLLR
SMG5 protein	37	0.0081	NLPPLR
		0.0062	NLPPLR
Plakophilin-1	36	0.00045	LDAEVPTR
Solute carrier family 35 member F5	35	0.0067	FTLSK
		0.015	FTLSK
Tripartite motif-containing protein 45	33	0.0042	HGDSVR

Glycosylation-dependent cell adhesion molecule 1	32	0.016	EQIVIR
Glutathione S-transferase P	32	0.0044	ASCLYGQLPK
Cathepsin D	31	0.0013	ISVNNVLPVFDNLMQQK
Pancreatic trypsin inhibitor	30	0.0017	AGLCQTFVYGGCR
Myozenin-2	26	0.019	VSIPR
GLIPR1-like protein 1	25	0.021	TAEAWAK
Elongation factor 1-alpha	24	0.0081	IGGIGTVPVGR

Supplementary Table 2: Protein identities, their probability scores (protein score and expect score) and peptide sequences (>95% confidence) in the raft fraction.

Protein name	Protein score	Expect score	Peptide sequence
Glial fibrillary acidic protein	42	0.00014	LALDIEIATYR
Phosphatidylinositol 3-kinase	24	0.052	HGDDLR
NADH dehydrogenase [ubiquinone] iron-sulfur protein 2, mitochondrial	27	0.021	LIEYK
Hexokinase	239	0.004	LALLQVR
		0.0079	ASGVEGTDVVK
		0.0039	YLSQIESDR
		0.053	FNTSDVSAIEK
		0.0000014	MVSGMYLGELVR
		0.0027	MISGMYLGEIVR
		0.0007	SANLVAATLGAILSR
		0.0012	SANLVAATLGAILSR
		0.00037	SANLVAATLGAILSR
		0.00000013	AAQLCGAGMAAVVDK
		0.00089	STPDGTENGDFLALDLGGTNFR
Hexokinase (Fragment)	87	0.0027	MISGMYLGEIVR
		0.00000013	AAQLCGAGMAAVVDK
Hexokinase-1	31	0.004	LALLQVR
		0.053	FNTSDVSAIEK
HK3 protein	27	0.0027	MISGMYLGEIVR
Alpha-fetoprotein	24	0.0098	YIYEIAR
Junction plakoglobin	223	0.00026	EGLLAIFK
		0.0023	LAEPSQLLK
		0.024	QEGLESVLK
		0.00014	LLNDEDPVVVTK
		0.00032	TMQNTSDLDTAR
		0.0036	HPEAEMAQNSVR
		0.0025	ALMGSPQLVAAVVR
		0.0000037	ALMGSPQLVAAVVR
		0.0059	LLNQPNQWPLVK
		0.0018	NEGTATYAAAVLFR
		0.00035	NEGTATYAAAVLFR
Catenin beta-1	46	0.00026	EGLLAIFK

Actin, cytoplasmic 1	149	0.0093	IIAPPER
		0.00017	AGFAGDDAPR
		0.0072	EITALAPSTMK
		0.0013	EITALAPSTMK
		0.0033	QEYDESGPSIVHR
		0.0017	VAPEEHPVLLTEAPLNPK
		0.0002	DLYANTVLSGGTTMYPGIADR
		0.00034	DLYANTVLSGGTTMYPGIADR
		0.0093	IIAPPER
		0.00017	AGFAGDDAPR
		0.0033	QEYDESGPSIVHR
		0.0017	VAPEEHPVLLTEAPLNPK
Actin, aortic smooth muscle	76	0.0093	IIAPPER
		0.00017	AGFAGDDAPR
		0.0072	EITALAPSTMK
		0.0013	EITALAPSTMK
ACTA2 protein	67	0.00017	AGFAGDDAPR
		0.0072	EITALAPSTMK
		0.0013	EITALAPSTMK
Actin (Fragment)	30	0.0093	IIAPPER
Beta actin (Fragment)	30	0.0017	VAPEEHPVLLTEAPLNPK
Solute carrier family 2 (Facilitated glucose transporter), member 3	108	0.016	FLLINR
		0.0017	QVTVLELFR
		0.002	AFEGQTQTGTR
		0.0000034	GPIMEMNSIQPTK
		0.0028	GPIMEMNSIQPTK
Solute carrier family 2, facilitated glucose transporter member 1	27	0.016	FLLINR
Desmoglein-1 OS=Bos taurus GN=DSG1 PE=4 SV=2	95	0.041	LADISLGK
		0.000013	IHSDCAANQQVTYR
		0.00055	ISGVGIDQPPYGIFVINQK
		0.00049	ISGVGIDQPPYGIFVINQK
Cathepsin D	68	0.000021	FDGILGMAYPR
		0.00089	ISVNNVLPVFDNLMQQK
		0.00089	ISVNNVLPVFDNLMQQK

Plakophilin-1	45	0.00049	LDAEVPTR
		0.015	AVQYLSSQDEK
PKP1 protein	25	0.015	AVQYLSSQDEK
14-3-3 protein zeta/delta	45	0.000064	SVTEQGAELSNEER
Heat shock protein beta-1	43	0.000084	LFDQAFGLPR
Elongation factor 1-alpha	42	0.0047	EAAEMGK
		0.0014	YYVTIIDAPGHR
		0.0014	YYVTIIDAPGHR
		0.0047	EAAEMGK
Pancreatic trypsin inhibitor	40	0.00016	AGLCQTFVYGGCR
Tripartite motif-containing protein 45	38	0.0015	HGDSVR
Heat shock 70 kDa protein 1	37	0.00045	VEIIANDQGNR
Hyaluronidase	36	0.004	STQNAALYVR
		0.006	TVFGGIPQLGNLK
Solute carrier family 2, facilitated glucose transporter member 5 (Fragment)	36	0.0011	AVGFISVLK
Serine/threonine-protein phosphatase (Fragment)	32	0.0052	VNAIESSAIR
Insulin-degrading enzyme	30	0.0016	ESLDDLTNLVVK
Annexin A2	29	0.0017	TNQELQEINR
Bromodomain, testis-specific	27	0.034	KAAIEK
Solute carrier family 35 member F5	27	0.018	FTLSK
Alpha S1 casein	27	0.0031	FFVAPFPEVFGK
Peroxiredoxin-2	25	0.025	QVTINDLPVGR
Nuclear receptor subfamily 2 group C member 1	25	0.01	LPALR
Very-long-chain (3R)-3-hydroxyacyl-CoA dehydratase	25	0.012	AGAGEASSSRR
Rab GDP dissociation inhibitor beta	24	0.0052	FVSISDLLVPK
SLA protein	24	0.0076	KSVSLMYSGSK

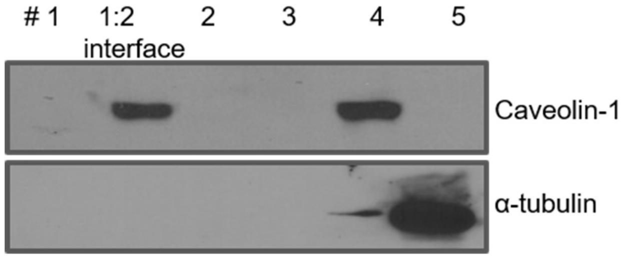

Supplementary Figure 1: Demonstration of α-tubulin (non-raft marker) showing successful separation of raft and non-raft fractions in sperm, despite the presence of caveolin-1 (classical raft marker) in both fractions. 1:2 interface correspond to the raft fraction which was collected at the 5–30% sucrose interface; 2 & 3 correspond to intermediate fractions; 4 & 5 correspond to non-raft fractions that was collected at the bottom of the sucrose gradient. This figure is reproduced with permission from Rajamanickam GD, Kastelic JP, Thundathil JC article on "Na/K-ATPase regulates bovine sperm capacitation through raft- and non-raft-mediated signaling mechanisms" published in Molecular Reproduction and Development 2017 Nov 84(11): 1168-1182.
